# Odontogenic Keratocyst With Moderate Epithelial Dysplasia: A Rare Entity

**DOI:** 10.7759/cureus.56702

**Published:** 2024-03-22

**Authors:** Vimalasubhashini Vivekbalamithran, Karthikeyan Ramalingam, Pratibha Ramani, Mahathi Neralla, Alden S Jason

**Affiliations:** 1 Oral Pathology and Microbiology, Saveetha Dental College and Hospitals, Saveetha Institute of Medical and Technical Sciences, Saveetha University, Chennai, IND; 2 Oncology, Saveetha Dental College and Hospitals, Saveetha Institute of Medical and Technical Sciences, Saveetha University, Chennai, IND; 3 Oral and Maxillofacial Surgery, Saveetha Dental College and Hospitals, Saveetha Institute of Medical and Technical Sciences, Saveetha University, Chennai, IND

**Keywords:** follow-up, reconstruction, fibula graft, surgery, condyle, biopsy, ramus, mandible, dysplasia, odontogenic keratocyst

## Abstract

We present a rare case of odontogenic keratocyst (OKC) with moderate epithelial dysplasia in a 47-year-old male patient. He presented with a history of pain and swelling on the right side of his face for the past three months. The radiograph revealed multi-locular radiolucency involving the ramus, coronoid process, and condylar process of the right mandible. We have discussed the cytology, incisional biopsy, radiological investigations, surgical management, reconstruction, excisional biopsy report, and follow-up of this rare entity.

## Introduction

Odontogenic keratocyst (OKC) has been a controversy due to its locally aggressive behavior and higher recurrence. Sporadic cases of malignant transformation are reported but very little is known about dysplasia in OKC [1-3]. Atypia of OKC lining is a rare entity that significantly influences treatment planning [4]. In this case report, we present a case of OKC that underwent cytology, incisional biopsy, and subsequent excision. We discuss the clinical findings, radiological features, surgical therapy, reconstruction, and follow-up information of an OKC with moderate epithelial dysplasia.

## Case presentation

A 47-year-old male patient reported to the outpatient department of Saveetha Dental College and Hospitals, Chennai, India with a complaint of swelling in the right lower jaw for the past few weeks. He had noticed sharp, intermittent pain in the right lower back teeth region for three months and a history of swelling on the right side of the mandible, which was slowly growing in size. Medical history and surgical history were noncontributory. He had undergone an uneventful dental extraction of the mobile lower tooth a few months prior. 

**Figure 1:**
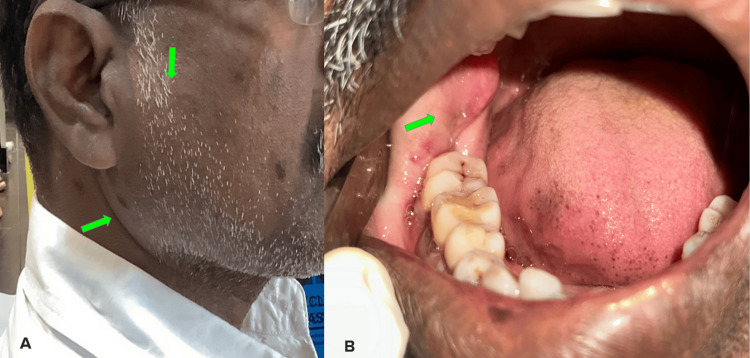
Clinical pictures showing the diffuse extra-oral swelling on the right side of the mandible (A) and diffuse intraoral swelling in the molar-ramus region (B) Green arrows indicate the swelling noted in the picture.

Extraoral swelling was seen on the right side of the face, which extended from 4 cm away from the corner of the mouth anteriorly and posteriorly 1 cm away from the ala-tragus. Superiorly, it extended below the zygomatic arch and inferiorly up to the submandibular region. A diffuse intraoral swelling was also noted in the retromolar region extending into the ramus and pterygomandibular raphe. On palpation, the swelling was soft to firm in consistency (Figure 1).

**Figure 2:**
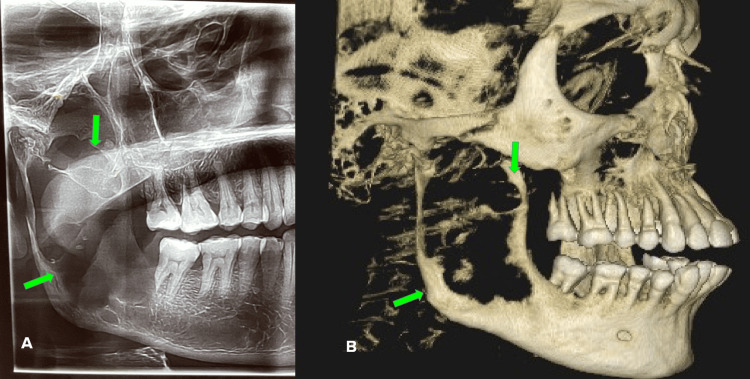
Radiograph revealing diffuse radiolucency involving the right mandibular ramus extending into the condyle along with perforation of cortical plates as seen in the orthopantomogram (A) and cone beam computed tomography (CBCT) (B) Green arrows indicate the osteolytic lesion of the right mandible.

Radiographs revealed an extensive osteolytic lesion involving the right mandibular ramus region. A cone beam computed tomography (CBCT) revealed well-defined unilocular radiolucency with expansion and breach in buccal, lingual cortical plates extending from the distal root of 47 to the neck of the right condylar head. Obliteration of the right inferior alveolar canal was noted along with morphological alteration of the right condyle (Figure 2).

Clinical diagnosis was made as OKC or ameloblastoma, and the patient was referred to the Oral Surgery Department for further investigations. 

**Figure 3:**
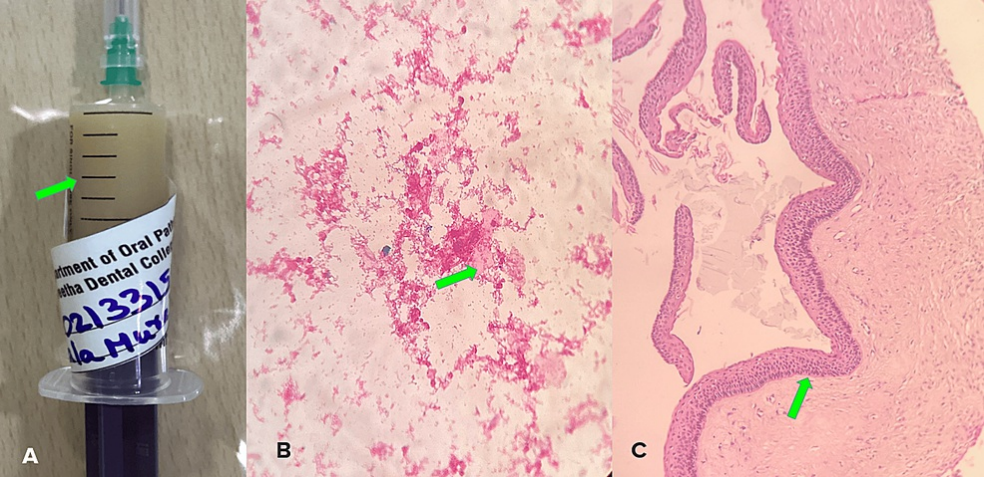
Aspirated sample (A), cytopathology (B), and histopathology (C) A - Syringe containing cream-like fluid aspirated from the lesion as indicated by a green arrow B - Photomicrograph of cytopathology showing few nucleated and anucleated squames in an eosinophilic background (H&E, 20x) as indicated by a green arrow C - Photomicrograph of incisional biopsy showing classic keratinized odontogenic epithelial lining with palisaded basal cells (H&E, 20x) as indicated by a green arrow

Aspiration revealed a creamy fluid that showed nucleated and anucleated squames with necrotic debris in cytopathology. An incisional biopsy was also performed and sent to the Department of Oral Pathology for processing. Histopathology revealed classical features of OKC with palisaded basal cells, parakeratinized odontogenic epithelial lining, flat interface, and few areas of epithelial detachment. It was diagnosed as OKC (Figure 3).

**Figure 4:**
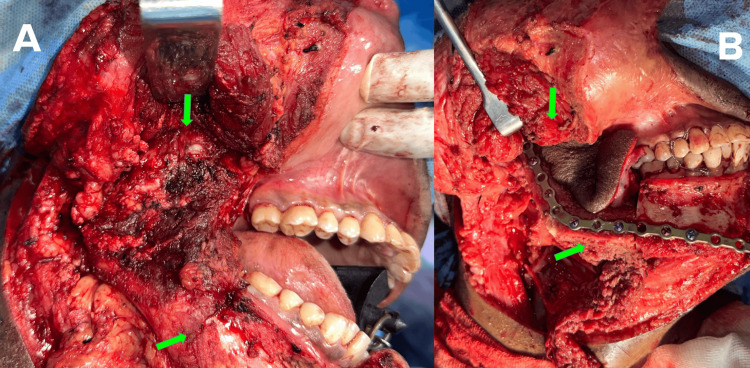
Intraoperative pictures A - Lesion involving the right mandibular ramus region before resection B - Reconstruction with free fibula graft and reconstruction plate

Hence, surgical resection of the involved mandibular ramus was performed along with enlarged lymph nodes in Levels IB and II. Free fibula reconstruction was also done along with a 2 mm reconstruction plate (Figure 4).

**Figure 5:**
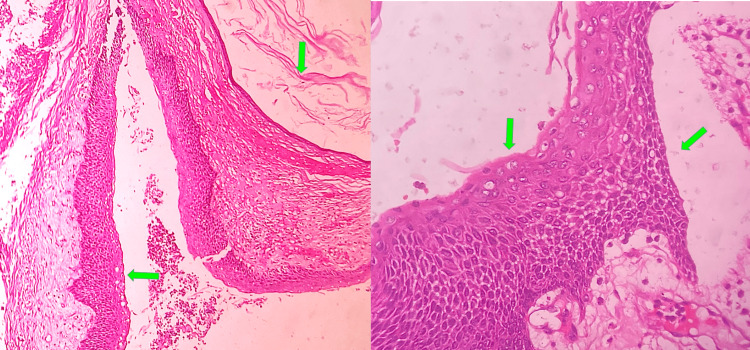
Photomicrograph of excisional biopsy showing odontogenic keratocyst with moderate epithelial dysplasia (H & E 20x, 40x) Green arrows indicating keratinization, keratin flakes, and cellular pleomorphism of the odontogenic epithelial lining.

Histopathological examination of excisional biopsy reported several areas of hyperchromatism, increased nuclear-cytoplasmic ratio, abnormal superficial mitosis, basal cell proliferation, and loss of cohesion, but the epithelial connective tissue interface was predominantly flat. The odontogenic epithelium was parakeratinized stratified squamous epithelium of uniform four-to-six-cell layer thickness with palisaded basal cells resembling tombstones. The epithelial-connective tissue interface was predominantly flat with few odontogenic cell rests and satellite cysts on the wall. Moderate chronic inflammatory cell infiltrate was also noted. Excised lymph nodes showed reactive hyperplasia (Figure 5).

**Figure 6:**
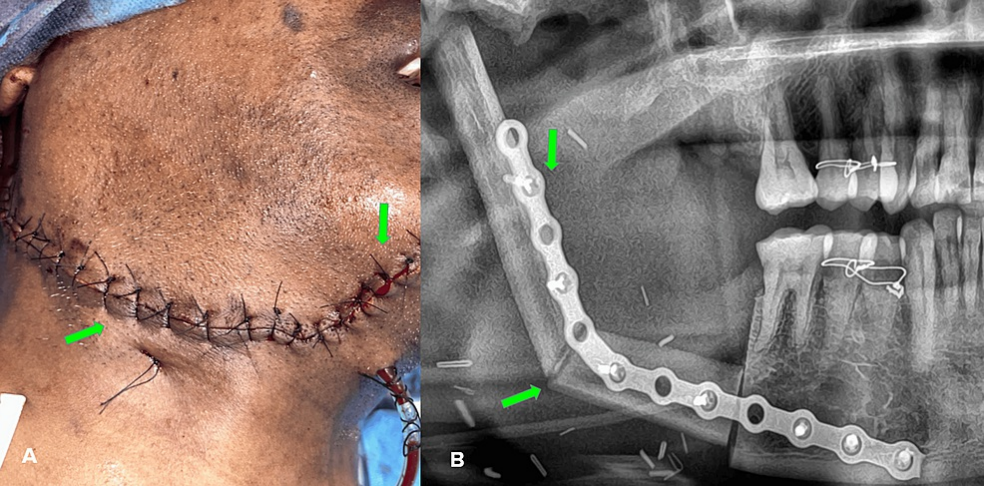
Follow-up images: clinical (A) and orthopantomogram (B) A - Clinical picture showing the surgical wound closed with sutures B - Orthopantomogram showing the fibula graft along with the reconstruction plate

The final diagnosis was given as OKC with moderate dysplasia, and the patient was instructed to be under strict follow-up. Wound healing was satisfactory on follow-up. Postoperative orthopantomograms (OPG) showed a partially edentulous upper and lower arch, generalized horizontal bone loss, and radiopaque surgical wires seen in the upper and lower arch relative to 14, 15, 24, 25, 34, 35, 44, and 45, discontinuity of the mandible on the right side with radiopaque surgical plates and screws (Figure 6).

The patient is under clinical and radiological follow-up to monitor recurrence and is disease-free after surgery.

## Discussion

OKC is a developmental odontogenic cyst per the latest WHO classification [1,2]. Epithelial dysplasia in OKC is a key indicator and has a 0.12% risk for malignant transformation [4-6]. The presence of dysplasia with long-term inflammation is a known risk factor for recurrence [4]. Elnouaem et al. [7] reported a malignant change of OKC in a child. Tamgadge et al. [8] reported that OKC showed higher mitotic activity and has an increased potential to change into squamous cell carcinoma. Cottom et al. [9] reported that sub-epithelial splitting, the position of mitotic figures, and sub-epithelial hyalinization can predict a higher recurrence rate. Jung et al. [10] reported that large OKC with multilocularity and conservative surgery have a higher recurrence. They have recommended a 10-year follow-up after any treatment of OKC.

OKC has been associated with PTCH1 mutations, allelic imbalance in 9p21, dysregulation of p53, increased Ki67, and epidermal growth factor pathway. The frequency of malignant transformation in odontogenic cysts was reported to be 0.01%-0.02% [5,10]. Proliferative markers, such as PCNA, Ki67, and p53, were expressed at higher levels in a keratocystic odontogenic tumor (KCOT). In addition to the mutation of PTCH genes, CDKN2A, TP53, MCC, CADM1, and FHIT mutations have been reported in OKC [4]. Ajayi et al. [6] performed argyrophilic nucleolar organizer region (AgNOR) scores and degree of epithelial dysplasia in odontogenic cysts (Appendix). They reported that 44.5% of OKC showed moderate epithelial dysplasia with nuclear pleomorphism being the most observed feature. Our case showed hyperchromatism, an increased nuclear-cytoplasmic ratio, abnormal superficial mitosis, basal cell proliferation, and loss of cohesion.

Many treatment methods have been attempted on OKC to reduce this recurrence, such as Carnoy’s solution, cryotherapy, and peripheral ostectomy [10]. OKC is mainly treated with enucleation with or without decompression [11]. Stoelinga has reported a treatment algorithm to avoid recurrences in OKC [12]. Jung et al. [10], Al-Moraissi et al. [13], and Oginni et al. [14] recommended en-bloc resection for reducing the recurrence of OKC. Our patient was also treated with surgical resection of the right mandible with reconstruction.

Target therapy and gene therapy for PTCH mutations and smooth end receptors may develop into a novel protocol to reduce OKC's occurrence, growth, and recurrence. Regular periodic radiographs are mandatory for monitoring purposes [10]. EGFR polymorphisms could play a role in future classifications of OKC [15]. Cai et al. recommended the generation of digital prognostic and diagnostic models to predict the behavior of OKC [16].

Due to the high recurrence rate of OKC, research has been directed toward identifying risk factors that may contribute to OKC recurrence. Although there have been reports of an increased proliferative potential in OKCs carrying PTCH1 truncation-causing mutations, it has been found that the expression of COX-2, bcl-2, PCNA, and p53 was not linked to OKC recurrence [16].

Table 1 shows the reported assessment of different odontogenic cysts for dysplastic features with immunohistochemical staining.

**Table 1:** Reported assessment of odontogenic cysts for dysplasia

Author Name	Type of cyst assessed	Findings	Dysplasia
Jalali et al. [5]	Odontogenic keratocyst	Nests of malignant epithelial cells seen in cohesion with the cyst lining.	Evident
Pandiar et al. [17]	Odontogenic keratocyst	Loss of p53 expression in the proliferative cyst lining.	Evident
Cox [18]	Odontogenic keratocyst	Strong basal cell staining and suprabasilar staining of p53.	Evident
Likhithaswamy et al. [4]	Odontogenic keratocyst	Focal areas showing basal cell budding with features such as loss of basal cell polarity, cellular and nuclear pleomorphism, nuclear hyperchromatism, altered nuclear-cytoplasmic ratio, dyskeratosis, and mitosis in the supra-basal layer.	Evident
Cox [18]	Odontogenic keratocyst	No basal cell staining and no suprabasilar staining of p53.	Not evident
Kalele et al. [4]	Odontogenic keratocyst	The majority lining showed features like hyperchromatism, basal cell hyperplasia, koilocytes with tiny satellite cysts.	Evident
Cox [18]	Odontogenic keratocyst	Weak basal cell staining and no suprabasilar staining of p53.	Not evident
Cox [18]	Orthokeratinized Odontogenic cyst	Strong basal cell staining and suprabasilar staining of p53.	Evident
Current case	Odontogenic keratocyst	Several areas of hyperchromatism, increased nuclear-cytoplasmic ratio, abnormal superficial mitosis, basal cell proliferation, and loss of cohesion but the epithelial connective tissue interface was predominantly flat.	Evident

## Conclusions

In this case report, we have discussed the detailed clinical presentation, cytology, biopsy, radiological examination, and surgical management of OKC, which appeared innocuous at the time of an incisional biopsy but turned out to be showing moderate epithelial dysplasia after excision. The patient has to be under strict clinical and radiological follow-up to monitor and avoid recurrence.

## Appendices

List of architectural disturbances (A) and the features scored (B) [6]

A. The Following Atypical Features Were Considered Features of Epithelial Dysplasia (Architectural Disturbances):

Irregular epithelial stratification

Hyperplasia of the basal layer

Drop-shaped rete ridges

Increased number of mitotic figures (at least two mitotic figures per high-power field, in a minimum of two different fields)

Loss of polarity of basal cells (this will be considered only in areas where the stratum basale is intact)

Increased nuclear-cytoplasmic ratio (at least five cells with this feature per high-power field, in a minimum of two different fields)

Nuclear polymorphism (at least ten abnormal cells per high-power field, in a minimum of two different fields)

Nuclear hyperchromatism (at least 10 hyperchromatic nuclei per high-power field, in a minimum of two different fields)

Enlarged nucleoli (at least 10 per high-power field, in a minimum of two different fields)

Keratinization of a single cell or cell group (at least two foci of keratinization per high-power field, in a minimum of two different fields)

Loss of intercellular adherence

B. Enumeration of Epithelial Dysplasia (Features Scored)

No dysplasia: A case was diagnosed as no dysplasia and scored 0 if no feature of dysplasia was seen and there was no evidence of disturbance in the epithelium.

Mild dysplasia: A case was evaluated as mild dysplasia and scored 1 if one or two features of dysplasia were observed and disturbance in the epithelium was limited to the lower third of the epithelium.

Moderate dysplasia: A case was evaluated as moderate dysplasia and scored 2 if three or four features of dysplasia were observed and disturbance in the epithelium extended up to two-thirds of the epithelium starting from the basal layer.

Severe dysplasia: A case was evaluated as severe dysplasia and scored 3 if features of dysplasia were seen numbered more than 4 and disturbance in the epithelium extends beyond two-thirds of the epithelium starting from the basal layer.
